# (Re)Construction of the body of transgender women: daily search for (in)satisfaction and care?

**DOI:** 10.1590/0034-7167-2021-0512

**Published:** 2022-08-08

**Authors:** Carle Porcino, Jeane Freitas de Oliveira, Maria Thereza Ávila Dantas Coelho, Dejeane de Oliveira Silva, Cleuma Sueli Santos Suto, Pablo Luiz Santos Couto, Helena Moraes Cortes, Antônio Marcos Tosoli Gomes

**Affiliations:** I Universidade Federal da Bahia. Salvador, Bahia, Brazil; II Universidade Estadual de Santa Cruz. Ilhéus, Bahia, Brazil; III Universidade do Estado da Bahia. Senhor do Bonfim, Bahia, Brazil; IV Centro de Ensino Superior de Guanambi. Guanambi, Bahia, Brazil; V Universidade Federal de Santa Catarina. Florianópolis, Santa Catarina, Brazil; VI Universidade do Estado do Rio de Janeiro. Rio de Janeiro, Rio de Janeiro, Brazil

**Keywords:** Transgender Persons, Free Association, Human Body, Nursing, Vulnerable Populations., Personas Transgénero, Asociación Libre, Cuerpo Humano, Enfermería, Poblaciones Vulnerables., Pessoas Transgênero, Associação Livre, Corpo Humano, Enfermagem, Populações Vulneráveis.

## Abstract

**Objective:**

To analyze the structure and contents of transgender women’s social representations of their bodies and body modification practices.

**Methods:**

Research conducted with 92 women using the Snowball technique. The data were collected using the free evocation of words technique and processed by the Evoc software, which organized the central and peripheral elements.

**Results:**

The representation of the real body includes two structuring aspects: one related to the need to adapt/modify the body conformation according to the self-reported gender, because of the dissatisfaction with the body itself; the second reveals the happiness/satisfaction considering the results obtained through the body modification/adaptation practices adopted in the transition.

**Final considerations:**

The body is constituted as a complex object and was represented by elements that reinforce the understanding of body modifications as needs, with a view to satisfaction, personal fulfillment, and care of one’s own body.

## RESUMO

**Objetivo:**

Analisar a estrutura e os conteúdos das representações sociais de mulheres transgêneras sobre o corpo e práticas de modificação corporal adotadas.

**Métodos:**

Pesquisa realizada com 92 mulheres mediante a técnica *Snowball*. Os dados foram coletados com auxílio da técnica de evocação livre de palavras e processadas pelo software Evoc, que organizou os elementos centrais e periféricos.

**Resultados:**

A representação do corpo real abarca dois aspectos estruturantes: um relativo à necessidade de adequar/modificar a conformação corporal consoante o gênero autorreferido, por causa da insatisfação com o próprio corpo; o segundo revela a felicidade/satisfação considerando os resultados obtidos por meio das práticas de modificações/adequações corporais adotadas na transição.

**Considerações finais:**

O corpo se constitui como um objeto complexo e foi representado por elementos que reforçam a compreensão das modificações corporais como necessidades, com vistas à satisfação, realização pessoal e cuidados com o próprio corpo.

## RESUMEN

**Objetivo:**

Analizar estructura y contenidos de las representaciones sociales de mujeres transgénero sobre el cuerpo y prácticas de modificación corporal adoptadas.

**Métodos:**

Investigación realizada con 92 mujeres mediante la técnica *Snowball*. Los datos fueron recolectados con auxilio de la técnica de evocación libre de palabras y procesadas por el software Evoc, que organizó los elementos centrales y periféricos.

**Resultados:**

La representación del cuerpo real engloba dos aspectos estructurantes: un relativo a la necesidad de adecuar/modificar la conformación corporal consonante el género autoinformado, por causa de la insatisfacción con el propio cuerpo; el segundo revela la felicidad/satisfacción considerando los resultados obtenidos por medio de las prácticas de modificaciones/adecuaciones corporales adoptadas en la transición.

**Consideraciones finales:**

El cuerpo se constituye como un objeto complejo y fue representado por elementos que refuerzan la comprensión de las modificaciones corporales como necesidades, con fin a la satisfacción, realización personal y cuidados con el propio cuerpo.

## INTRODUCTION

Body modification practices are resources developed by biomedicine, to which transgender people can resort to “fit” and/or “conform” their bodies. The goal is to achieve body materiality and personal satisfaction linked to what they conceive as the ideal body, with possibilities of re-signifying the (inter)subjective aspects involved in this process.

Transgender people are understood as those who psychosocially do not identify with the exclusive gender belonging imposed on them at birth^(1-2)^, which takes into consideration the genital anatomical conformation, relying on the social convention established by the cis-hetero norm^(1,3)^. In this way, the process of (self)recognition based on self-determined gender can occur both in childhood and adulthood^(4)^, given that this is an experience lived in a unique way by each person throughout life. It is noteworthy that gender self-determination is a right that is not always fully recognized^(5)^, although the Yogyakarta Principles^(6)^ that every person has the right to be legally recognized, according to the gender he or she identifies with and feels he or she belongs to, without being subjected to body modification techniques and/or unwanted surgical interventions.

Thus, even if one and/or another body modification technique is adopted by transgender people, taking into account the desire, personal fulfillment, and available resources, the health professional must recognize the autonomy, protagonism, and legitimacy of the person before this process. Therefore, it is essential to reflect on the mistake that is still made when considering a transgender person as necessarily synonymous with a surgically or re-designated person^(7)^. In this respect, one cannot lose sight of the meaning of the body and (bio)medicine as a bio-political reality and strategy, since society’s control over individuals will not only occur through consciousness and ideology, but also through the body(ies), with the body(ies), and over the body(ies)^(8)^. Although these are marked by social and symbolic inscriptions, which are materialized by the person itself^(9)^, it is in and through the body that the processes of self-affirmation and/or transgression of the regulatory norms imposed by the system are expressed^(2)^, enabling the existence of dissident bodies.

In this aspect, it is relevant to highlight that, in Brazil, research developed with the participation of transgender and transvestite women, undertaken by transgender researchers, is still incipient^(4,10-12)^, since the largest number of productions in which this population segment was the object of study came from research conducted by cisgender people^(13-15)^. However, this fact does not constitute a prejudice, because the importance of these studies on gender dissidences and transgenerities that contributed to the construction of knowledge within this field of knowledge is recognized. However, a study led by a transgender woman with other cisgender women can make this contribution even more expressive given the singularities, subjectivities and construction of (trans)episteme through exchanges and interactions that involve and cross the body(ies) in this process, as is the case of this research.

Thus, the body is a privileged object for studies using the Social Representations Theory (SRT)^(16-17)^ by enabling interaction/exchange between individuals and groups as they objectify and anchor the notions and images directed toward material and symbolic practices in relation to the corresponding object^(17)^. In this sense, they provide access to knowledge, as they guide and ground the care practices. Therefore, understanding the shared knowledge present in the social thinking of transgender women and how these influence attitudes and behaviors, especially regarding their own bodies and body modification practices, allows us to reflect on the health care provided to this population segment. Therefore, in relation to the demands arising from gender transition and health needs, it is essential to consider the knowledge they have built about and from themselves, as a form of self-care and health promotion^(18)^.

It should be noted that this is an aspect that will subsidize both the recognition of the right to gender self-determination by health professionals and the identification of possible factors that interfere with the planning and dispensation of comprehensive and equitable care. In this sense, the observance of these actions contributes to the reduction of damage and minimization of health risks, resulting from non-assistance and barriers faced in access to health services by transgender women.

## OBJECTIVE

To analyze the structure and contents of transgender women’s social representations of their bodies and body modification practices.

## METHODS

### Ethical aspects

The project was registered in the *Plataforma Brasil*, reviewed and approved by the Ethics Committee on Human Research of the School of Nursing of the Federal University of Bahia and by the Ethics Committee on Human Research of the Secretary of Health of the State of Bahia, respecting the ethical precepts^(19)^. As a way of preserving anonymity, the following coding was used (P-1, P-2... P-92), considering the numerical order of the answers to the free evocation of words technique, plus the age of the person and the information on the age at which he/she first used hormones.

### Theoretical and methodological framework

This research is based on SRT, proposed by Serge Moscovici^(20)^, focusing on the structural approach developed by Jean-Claude Abric^(21)^, also known as the Central Core Theory (CCT). According to this approach, a representation has two components: content and structure. It also has four primary functions: the knowing function, which allows for the understanding and explanation of reality; the identity function, which provides protection to group specificity; the orienting function, which guides behaviors and social practices; and the justifying function, which provides justification for taking a position^(21)^.

Social representations are conceived as forms of practical knowledge that imply an intrinsic relationship between a subject and an object, symbolized through representation and interpreted by the subject as it refers to the object itself^(16)^. They also constitute a particular kind of knowledge, whose specificity lies in the social processes in which they are produced^(21)^. They also have the function of recognizing and elaborating behaviors among subjects, through the interactions they establish with the context, as they recognize and identify themselves as part of a social group^(20)^. In this sense, research in social representations allows the apprehension of history in its construction process^(16)^ and demands a simultaneous articulation based on three levels of analysis: culture, interaction, and individual position^(22)^.

### Type of study

This is a descriptive and exploratory study, with a qualitative approach, which was interested in the common sense and worldview that individuals and groups use to understand the dynamics of previous experiences, based on social interactions, focusing on the identification of social practices and their determinants^(21)^. In order to ensure the quality and rigor of the research, the guidelines of the Consolidated criteria for reporting qualitative research (COREQ) checklist were followed^(23)^.

### Study scenario

The study was developed in the city of Salvador, state of Bahia (BA), since two services that serve the transgender population in the state are located in this capital, one of them being qualified by the Brazilian Ministry of Health. These aspects allowed access to a larger number of transgender women. Sampling was carried out using the consecutive recruitment strategy Snowball^(24)^ to reach the participants. The adoption of this technique occurred due to the absence of information on how many transgender people exist, or how many have health care demands. For this, invitations were made through telephone contacts, with the first five seeds, after the indication of a representative of the Social Organized Movement of Trans People from Salvador, Bahia, Brazil. In this way, each participant was asked to indicate another transgender woman, and so on, until the end of the data production. The data collection did not happen in a determined place, but the day, time and place were previously agreed with each person.

### Source of data

Data was produced by the participation of 92 transgender women. Inclusion criteria were: transgender women based on self-declaration and self-determination of gender; aged 18 years or older. We excluded those living outside Salvador, at the time of data collection and who could not interact with the researcher due to the pandemic situation of COVID-19 and/or were in home recovery due to some surgical procedure.

### Collecting and organizing data

The production of the empirical data was carried out individually by the researcher herself in a face-to-face manner with each participant and occurred in the months of December 2019 to June 2020. To this end, the free word evocation technique was applied, and participants were asked to speak the first five words and/or short expressions that immediately came to mind upon hearing the inductive term “The real body for me is...”. They were then asked to indicate the most important term and justify their choice^(25)^. Then, in order to know the profile of the social group, a questionnaire was applied with open and closed questions about socio-demographic aspects, health conditions, body modification practices adopted, hormone use, and level of satisfaction with the practices adopted. It should be noted that this instrument was applied after the free evocation of words technique, to avoid possible influences on the data produced.

### Data analysis

For the analysis of socio-demographic data and body shaping practices, descriptive statistics were used, with the help of the Microsoft Excel spreadsheet editor. The evocations were processed by the software “*Ensemble de programmes permettant l’analyse des évocations” (EVOC)*, version 2005^(26)^, which organizes the content of the representational field and its structure by constructing the four-box frame^(27)^.

In the upper left quadrant are the elements that make up the probable central nucleus because they have the highest frequencies and were most readily evoked. The upper right quadrant, called the first periphery, contains elements with frequencies even higher than the central nucleus, but evoked later. The lower left quadrant, the contrast zone, is considered the second most important of the representational structure, because, besides conferring quality to the central elements, it can also indicate the formation of a subgroup. The lower right quadrant, also known as the second periphery, groups the terms and expressions that are not so important for the group about the representational object, but that are reflected in everyday life^(21,27)^.

The justifications attributed to the terms classified as most important were used in order to contextualize and reveal the semantics of the evocations and the relationship between the elements present in the four-box frame. Similarity analysis was employed in order to reach the overall structure of the representation. The calculation was performed considering the number of co-occurrences between two evocations, divided by the number of participants, which results in the similarity index, which can be observed in the maximum similarity tree. Through this technique, it is not possible to confirm the centrality of these elements, but to point out those that are considered central for the group^(28-30)^, taking into account the social, historical, ideological aspects and the norms that guide the social group^(21)^.

## RESULTS

Among the 92 participants, 63% were religious; 79% were between 18 and 39 years old; 58% were from Salvador; 86% were of the race/color brown/black; 97% were single; 84% declared themselves heterosexual; 46% had incomplete high school; 38% worked informally (10% had formal jobs; 9% were fired from formal jobs due to the pandemic of COVID 19; 21% lived exclusively from sex work; 14% had formal/informal jobs and complemented their income with sex work); 62% had an income of up to two minimum wages, 27% between two and four, and 11% over four minimum wages; 87% accessed exclusively the services of the Unified Health System (UHS); 9% had access to the supplementary network and to UHS; 4% accessed exclusively the supplementary network; 22% accessed hormone therapy in the Trans-sexualization Process (TrPr) in UHS; and 69% reported having suffered some type of discrimination in UHS.

Regarding the practices adopted in the transition towards body conformation, 99% have already performed some clinical and/or surgical procedure to conform their body; 71% considered body modification practices as a very important aspect; and 53% were satisfied with the results achieved.

**Figure 1:**
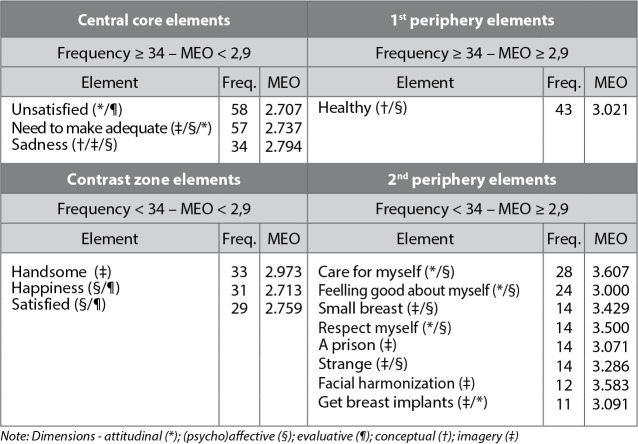
Four-box table for the inductive term “The real body for me is...”: central and peripheral elements of social representations of transgender women (N = 92), Salvador, Bahia, Brazil, 2021

The text corpus built based on the evocations arising from the inductive term “The real body for me is...” totaled 460 words, among which 23 were different. With this value, a minimum frequency of 11 was established and a mean frequency of 34 was calculated, whose mean evocation order (MEO) was 2.9. Using these parameters, the table of four boxes was prepared (Figure 1), which reveals the central elements (upper left quadrant) and peripheral elements (the other quadrants).

In the upper left quadrant, “must fit”, “unsatisfied” and “sadness” indicate the possible central elements of the representation, which refer to the conceptual, imagery, affective and attitudinal/evaluative dimensions.

*Dissatisfied is a term that constantly reflects what I feel when I look in the mirror and want to change it, but cannot immediately. I am dissatisfied because my current body does not reflect the correspondence between two aspects of my person, the physical and the spiritual.* (P-1, age 25, used hormone for the first time at age 20)

*The term “it is necessary to adequate” is the most important since it reflects the correction of existing negative aspects in a general sense and says in a generalized way this whole process of adequacy that I seek.* (P-14, age 22, used hormone for the first time at age 19)

*As we are not born with the dreamed body, adaptation ends up being the way out, because when you have money, you can pay to have the body you want* [...] *unfortunately, this is not my case* [...] *this is a situation that makes me very sad.* (P-9, 33 years old, used hormone for the first time at age 20)

In the upper right quadrant is the term “healthy”, which refers to the conceptual and evaluative dimensions.

*Because a body that is not healthy, a person may not be able to modify the parts he or she desires. Although my body is harmonious, I want to put in a 300 ml prosthesis to give it a little volume.* (P-12, 38, used hormone for the first time at age 22)

The elements “handsome”, “happiness” and “satisfied” present in the lower left quadrant seem to reinforce the imagetic, (psycho)affective and evaluative dimensions of the representation in its probable central core.

*I am satisfied and I think my body is beautiful because it is the body I have always dreamed of. I did the face, the chest, the buttocks, the hips, and genital fitness.* (P-69, 59, used hormone for the first time at age 19)

*Happiness is the word that defines how I really feel after the changes I made to my body.* (P-79, 51, used hormone for the first time at age 19)

In the lower right quadrant, the terms “take care of myself” and “feeling good about myself” stand out, expressing the attitudinal and (psycho)affective dimensions related to taking a stand and autonomy over one’s own life. On the other hand, “small breast”, “respect for myself”, “a prison”, “strange”, “facial harmonization”, and “get breast implants” point to the imagery, evaluative, attitudinal, and (psycho)affective dimensions.

*Because, if we are not careful with our body, our health can be compromised and it will be impossible to fulfill our dreams and desires.* (P-54, 27, used hormone for the first time at age 26)

*This situation* [small breasts] *bothers me a lot because the hormone I take has not yet had the effect I want. Having a breast that fits our body and the psychological image we have of ourselves makes us happier.* (P 11, age 29, used hormone for the first time at age 19)

**Figure 2:**
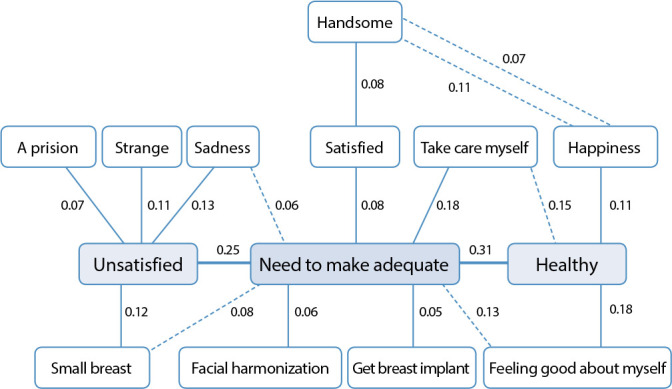
Maximum tree of the similarity analysis of the social representations of transgender women (N = 92), for the inductive term “The real body for me is...”, Salvador, Bahia, Brazil, 2021

Figure 2 shows the graphical representation of the maximum similarity tree, in which it is possible to observe the co-occurrences calculated through the frequency analysis, resulting from the connection between the terms. For the inductive term “The real body for me is...”, among the 92 participants, 90 simultaneously evoked two or more words among those that composed the four box frame.

In the body modification practices adopted by the participants, the following resources were used: the use of hormones, the performance of surgical procedures, and the application of Industrial Liquid Silicone (ILS). Here is a description of each one:

- Use of hormones (hormonization) on their own, here referred to as “unassisted transition” and/or “unassisted”, based on knowledge socialized by and among peers was referred to by 94%; for 63%, hormonizing is a very important aspect, while 47% were satisfied with the effects produced by the use of hormones in the body; the formulation combined estrogen and progesterone, used through intramuscular, oral, patch and transdermal gel routes with “Perlutan^®^”, “Climene^®^”, “Natifa^®^”, “Cicloprimogyna^®^”, “Evra^®^” and “Sandrena^®^” standing out. The use of the injectable presentation, for 54% of the participants, was made by a friend and/or acquaintance, 17% by self-application, 15% by a pharmacy employee and 14% by a health professional. The age range for first-time hormone use was from 8 years to 36 years of age. The use of blockers in oral presentation was reported by 9%, most notably: “Cyproterone Acetate^®^” (3), “Androcur^®^” (3) and Spironolactone^®^ (5);
- Surgical procedures performed by participants: laser facial hair removal (31), bilateral breast implantation (26), rhinoplasty (5), sex reassignment surgery (SRS) (3), facial feminization (2), liposculpture (2), augmentation mastoplasty (2), frontoplasty (1), reduction cheiloplasty (1), and mentoplasty (1); the degree of satisfaction with the practices adopted ranged from “satisfied,” “very satisfied,” and “completely satisfied”;
- ILS: was used by 40%; among them, 30% had complications resulting from its use; the lowest age at which the first application occurred was 19 years old and the highest, 40 years old; the predilection for part of the body was: buttocks, chest, hips, face and lips; the volume of ILS used varied from 20 ml to 10 liters; among those who used this technique, 22% were dissatisfied, 62% satisfied, 5% very satisfied and 11% completely satisfied; 30% had complications resulting from its use, one case being immediately after the application and the others between 5 and 10 years after the first application; 91% sought medical care at UHS, but only 18% had the situation resolved.

## DISCUSSION

With this study, it was possible to access the symbolic and imaginary constructions about the real body among transgender women. The analyzed meanings revealed the complexity that goes through the body dimension regarding the gender transition for this group. Thus, the social representations built by the participants, arising from their needs, experiences, and lived experiences, are materialized in the behaviors through speeches, ideas, and images under the aegis of two sources: the first is related to the set of knowledge that emerges from the traditions and experiences shared through the interaction in the group itself; and the second refers to the constructions and/or mental images, to the knowledge and wisdom that underlie everyday life^(20)^.

The elements allocated in the central nucleus, “dissatisfied”, “sadness”, and “it is necessary to adapt”, in the representational field under analysis, refer both to the functional and normative character. These elements, due to their hierarchies - most important for the group - have considerable symbolic value in relation to the represented object, because they concretize the social representations in social life and in the field of practical actions. Based on the TNC, the normative character is related to the value system of the group investigated, while the functional character favors the construction of the representation in the composition of the central nucleus, by grouping the most important elements for the realization and justification in taking a position^(21)^.

The “dissatisfaction” in relation to one’s own body, based on self-image and the need to adopt some practice of body modification, is present in the representations of the real body, given the idea of incompleteness that is anchored in the feeling of “sadness”. Such aspects were observed among younger, middle-aged women, with less education, who worked informally and had an income of up to two minimum wages. It is noteworthy that the constitution of the body, in which subjectivity is inscribed, will occur through constant changes by means of individual and social representations, since it is a private and social object^(31)^.

The term “sadness” contains the conceptual, (psycho)affective and evaluative dimensions associated with the element “dissatisfied” and refers to the dissatisfaction related to the conformation of the body, due to the absence of body materiality, since it is a process of self-identification. The expression “it is necessary to adapt” mentions the imagetic dimension, but also alludes to the (psycho)affective and attitudinal dimensions, given the evaluative character linked to taking a position, regarding the adoption of some practice to modify the body conformation, structuring the social representations about the real body. However, the practices of body modifications and their relationship with illness and health promotion, with a focus on self-care techniques^(18)^, are constructed through individual experiences and processes of peer sociability.

The peripheral elements are distributed in the three other quadrants and allow us to perceive several meanings attributed by the participants to the social representation of the real body. In the upper right quadrant, the term “healthy”, in terms of frequency, occupies the third position in the whole picture, objectifying the importance and maintenance of health in a consensual way in the representational field. It is evident that, for the group investigated, the importance of having health and/or a healthy body is an essential condition for the possible desired clinical and/or surgical changes to be materialized.

Most participants reported having suffered discrimination in access to health services, which can be configured as barriers to health maintenance and to an assisted transition. Thus, access to health as a basic human right is far removed from reality^(3,32-34)^. In spite of the fact that the specialized services for this segment are perceived as more qualified, the reception and assistance are not always free of prejudice and discrimination^(34-36)^. Thus, the quality of the care provided can be compromised and constitute a disservice, especially due to structural problems in the UHS and the lack of debates, in health training, about the health demands of transgender people^(3,33,36-37)^.

In the lower left quadrant, the terms “beautiful”, “happiness” and “satisfied” reflect variations of the group itself - of judgments, knowledge, value hierarchies, financial resources available for access to supplementary health care, age group and/or because they have already made body modifications that are possible and considered important to them -, pointing to signs of transition in their structure^(21)^. In this case, feeling beautiful, happy and satisfied with one’s own body, considering the modifications undertaken, may contribute not only to attitudes of self-care and increased esteem, but also to the reduction of risks and harm to health, in view of the experiences they share with each other.

The terms “taking care of myself”, “well-being with myself”, “small breast”, “respect for myself”, “a prison”, “strange”, “facial harmonization”, and “put the breast” present in the lower right quadrant portray a strong representation not only of the body, but also of life, which seems essential to achieve the idealized and desired body materiality - in terms of femininity, according to the self-reported gender. As for satisfaction, this representation was elaborated mainly by women who were satisfied with the body modifications performed. Such aspects connotes what they think is best for themselves, since they reaffirm the importance of adopting these practices both for personal satisfaction and for social recognition in daily life, an essential role in adaptation, in view of the evolutions of the context in which these representations emerge.

The element that most established connections in the maximum similarity tree (Figure 2) was “it is necessary to adapt” and “unsatisfied” and “healthy”. Thus, considering that the connectivity index established between the terms is a criterion for indicating centrality, it is possible to infer that these terms form the central core of the representation^(30)^.

The literature^(5,11-12)^ points out that the use of hormones on their own - hormonization - is common due to the barriers faced in accessing health services. While part (22%) of the participants accessed TrPr for hormone therapy, the surgical procedures performed with a view to body materialization were funded at their own expense, either by the absence of coverage or because of barriers in access to these services within the UHS^(5,15,33)^. It should be noted that SRT, facial feminization and frontoplasty were performed by Thai and Italian professionals, since the implementation of TrPr dates back to the last decade and the participants underwent these procedures more than 15 years ago. Regarding facial feminization and frontoplasty, both are still not funded by UHS, which leads transgender women who have financial resources to travel in and out of Brazil for these procedures.

The application of ILS is the only technique that is not performed by a health professional. Usually, this procedure is done by a transgender woman or transvestite, called a “pumper”^(38)^, which has social recognition within this population segment. In this sense, similar findings related to the use of ILS with the objective of giving the desired body materiality, more quickly, were also pointed out in the results of another study^(36)^. In this study, the minimum age of use was 19 years and the maximum, 40 years.

The use of ILS can cause risks and damage to health, such as: local inflammatory process, abscess, siliconoma, deformity, tissue necrosis, pulmonary embolism, migration to other parts of the body and, in its most severe form, death^(36,39)^. Thus, it is necessary to have discussions about the difficulties faced daily by transgender women regarding the care of their health demands and needs; and about the challenges faced by health professionals when receiving and planning care for this segment, considering that the discussion about transgender bodies is still incipient in the health field^(3,37)^.

Based on the results obtained, it is observed how emergent the use of these resources is for the social group, considering that the body is the space in which values, beliefs, attitudes, and behaviors are constructed and re-signified. In this sense, it is fundamental to consider some aspects, such as: motivation, desire, accessibility, available resources, and personal satisfaction. Moreover, this position statement, to some extent, can influence the construction of knowledge that emerges and is reified in the social environment, because it is developed through social (inter)relations and interactions among peers, which were and/or are incorporated into the knowledge available in the discourses of the group.

Moreover, new concepts, images, attitudes, and/or practices can be attributed to the represented object, taking into account the relational dynamics of the social group with this object. From this angle, the real body is an essential element in the transition because it is in it and through it that the dreamed, idealized, and desired changes will take place. It is through this corporal materiality that the processes of construction, (re)invention, and self-care are undertaken. Regarding them, each term evoked had its notoriety, given the complexity of the daily confrontations of transgender people, considering their demands and specificities, when seeking health services^(3-4,33-35)^.

### Study limitations

The limitation of this study stems from the participation of transgender women from a single municipality in Bahia, which does not allow advancing the results to other scenarios. However, even with the COVID-19 pandemic, it was possible to access an expressive number of participants in order to qualify the research results. Even with the impact of the pandemic of COVID-19 and its measures of social distancing and other health recommendations, the data collection stage was carried out in person.

### Contributions to the field of Nursing, Health or Public Policy

The relevance of this study lies in the importance of presenting the social representations of transgender women about the body and practices adopted in conforming the body, understood as a situated and declarative knowledge that takes into account the knowledge of common sense. In this aspect, Nursing and the other professional categories in the health area have a fundamental role with regard to respect for the use of the social name and gender self-determination, especially in the context of the UHS, since 69% of the participants reported having suffered some type of discrimination. The observance of these aspects is essential for us to recognize them in their entirety and provide a reception and quality care that addresses their real needs, respecting the autonomy of Trans people in order to reduce damage and health risks.

## FINAL CONSIDERATIONS

Based on the results presented, it is noted that social representations about the real body gain expression and visibility with the experiences undertaken by the participants to achieve the desired body materiality and consequent social recognition by others, considering the claimed gender. In this regard, we understand that the access to an “assisted transition” through the provision of quality services, effective public policies, professionals committed to comprehensive care, and extended and sensitive listening to the (trans)specificities of this segment potentiates the emancipatory care. We understand that the difficulties evidenced by the interviewees reflect the barriers faced by transgender women in accessing services and meeting their health needs. Encouraging managers to discuss this issue in technical and higher education in health is pertinent, since everyday experiences have a preponderant role in the processes involving health and disease.

## SUPPLEMENTARY MATERIAL

https://doi.org/10.48331/scielodata.OH38RA

0034-7167-reben-75-06-e20210512-sup01

0034-7167-reben-75-06-e20210512-sup02
