# Supplementary material for: (Re)Construction of the body of transgender women: daily search for (in)satisfaction and care?
Source: Rev Bras Enferm. 2022 Aug 8;75(6):e20210512. doi: 10.1590/0034-7167-2021-0512 (PMC9749498; doi:10.1590/0034-7167-2021-0512)
Supplement: 0034-7167-reben-75-06-e20210512-sup01 [file 0034-7167-reben-75-06-e20210512-sup01.pdf]

CÁLCULO PARA A ANÁLISE DE SIMILITUDE															
	Insatisfeita	É preciso adequar	Tristeza	Saudável	Bonito	Felicidade	Satisfeita	Cuidar de mim	Bem-estar comigo mesma	Peito pequeno	Respeito comigo mesma	Uma prisão	Estranho	Harmonização facial	Colocar o peito
Insatisfeita		23	12	18	8	0	4	4	3	11	2	7	10	6	5
É preciso adequar	0,25		12	28	7	8	3	17	12	8	2	6	2	6	5
Tristeza	0,13	0,13		9	3	0	1	2	3	2	0	5	5	1	3
Saudável	0,20	0,31	0,10		8	10	7	14	17	7	3	3	3	2	5
Bonito	0,08	0,07	0,03	0,08		7	3	4	6	0	3	3	2	1	2
Felicidade	0	0,08	0	0,11	0,07		10	8	8	0	5	4	0	2	1
Satisfeita	0,04	0,03	0,01	0,07	0,03	0,11		4	8	0	3	2	2	1	0
Cuidar de mim	0,04	0,18	0,02	0,15	0,04	0,08	0,04		10	1	3	0	0	2	4
Bem-estar comigo mesma	0,03	0,13	0,03	0,18	0,06	0,08	0,08	0,11		2	2	1	0	2	0
Peito pequeno	0,12	0,08	0,02	0,07	0	0	0	0,01	0,02		0	0	2	4	0
Respeito comigo mesma	0,02	0,02	0	0,03	0,03	0,05	0,03	0,03	0,02	0		1	0	0	2
Uma prisão	0,07	0,06	0,05	0,03	0,03	0,04	0,02	0	0,01	0	0,01		4	1	0
Estranho	0,11	0,02	0,05	0,03	0,02	0	0,02	0	0	0,02	0	0,04		1	0
Harmonização facial	0,06	0,06	0,01	0,02	0,01	0,02	0,01	0,02	0,02	0,04	0	0,01	0,01		0
Colocar o peito	0,05	0,05	0,03	0,05	0,02	0,01	0	0	0	0	0,02	0	0	0	
