# Supplementary material for: (Re)Construction of the body of transgender women: daily search for (in)satisfaction and care?
Source: Rev Bras Enferm. 2022 Aug 8;75(6):e20210512. doi: 10.1590/0034-7167-2021-0512 (PMC9749498; doi:10.1590/0034-7167-2021-0512)
Supplement: 0034-7167-reben-75-06-e20210512-sup02 [file 0034-7167-reben-75-06-e20210512-sup02.pdf]

001; 1 estranho 2 insatisfeita 3 satisfeita 4 estranho 5* uma-prisao
002; 1* bem-estar-comigo-mesma 2 felicidade 3 cuidar-de-mim 4 cuidar-de-mim 5 bonito
003; 1* saudavel 2 bonito 3 bem-estar-comigo-mesma 4 cuidar-de-mim 5 felicidade
004; 1 bonito 2 insatisfeita 3* e-preciso-adequar 4 saudavel 5 viver
005; 1 felicidade 2 bem-estar-comigo-mesma 3* satisfeita 4 saudavel 5 satisfeita
006; 1* uma-prisao 2 insatisfeita 3 insatisfeita 4 estranho 5 saudavel
007; 1* e-preciso-adequar 2 viver 3 felicidade 4 bonito 5 saudavel
008; 1* saudavel 2 satisfeita 3 bonito 4 bem-estar-comigo-mesma 5 insatisfeita
009; 1 insatisfeita 2 bunda-pequena 3 peito-pequeno 4* e-preciso-adequar 5 saudavel
010; 1 felicidade 2* uma-prisao 3 uma-prisao 4 saudavel 5 e-preciso-adequar
011; 1* peito-pequeno 2 insatisfeita 3 tristeza 4 tristeza 5 insatisfeita
012; 1 bem-estar-comigo-mesma 2 harmonizacao-facial 3 e-preciso-adequar 4* saudavel 5 peito-pequeno
013; 1* colocar-o-peito 2 tristeza 3 tristeza 4 insatisfeita 5 saudavel
014; 1 tristeza 2 tristeza 3* e-preciso-adequar 4 satisfeita 5 estranho
015; 1 saudavel 2* e-preciso-adequar 3 insatisfeita 4 tristeza 5 tristeza
016; 1 insatisfeita 2 e-preciso-adequar 3 e-preciso-adequar 4* saudavel 5 peito-pequeno
017; 1 e-preciso-adequar 2 cuidar-de-mim 3* insatisfeita 4 e-preciso-adequar 5 colocar-o-peito
018; 1* e-preciso-adequar 2 bonito 3 saudavel 4 cuidar-de-mim 5 felicidade
019; 1 insatisfeita 2 saudavel 3 insatisfeita 4 estranho 5* peito-pequeno
020; 1* insatisfeita 2 estranho 3 uma-prisao 4 tristeza 5 insatisfeita
021; 1 peito-pequeno 2 harmonizacao-facial 3 insatisfeita 4 insatisfeita 5* insatisfeita
022; 1 insatisfeita 2 tristeza 3 tristeza 4 insatisfeita 5* estranho
023; 1 bem-estar-comigo-mesma 2* saudavel 3 esta-velho 4 cuidar-de-mim 5 felicidade
024; 1 medo-de-envelhecer 2 tristeza 3 tristeza 4* e-preciso-adequar 5 insatisfeita
025; 1 felicidade 2 satisfeita 3 satisfeita 4 saudavel 5* e-preciso-adequar
026; 1 tristeza 2* insatisfeita 3 saudavel 4 e-preciso-adequar 5 bunda-pequena
027; 1 uma-prisao 2 tristeza 3* e-preciso-adequar 4 estranho 5 insatisfeita
028; 1* saudavel 2 e-preciso-adequar 3 medo-de-envelhecer 4 insatisfeita 5 viver
029; 1 felicidade 2 bonito 3 bem-estar-comigo-mesma 4* e-preciso-adequar 5 uma-prisao
030; 1 bonito 2 tristeza 3* insatisfeita 4 tristeza 5 saudavel
031; 1 insatisfeita 2 tristeza 3 e-preciso-adequar 4* e-preciso-adequar 5 cuidar-de-mim
032; 1* insatisfeita 2 bonito 3 insatisfeita 4 estranho 5 peito-pequeno
033; 1 e-preciso-adequar 2* bunda-pequena 3 insatisfeita 4 peito-pequeno 5 saudavel
034; 1 estranho 2 insatisfeita 3* estranho 4 estranho 5 harmonizacao-facial
035; 1 e-preciso-adequar 2* esta-velho 3 insatisfeita 4 bunda-pequena 5 peito-pequeno
036; 1 bonito 2 insatisfeita 3 satisfeita 4 bonito 5 bonito
037; 1 felicidade 2 harmonizacao-facial 3 bem-estar-comigo-mesma 4 cuidar-de-mim 5* e-preciso-adequar
038; 1 saudavel 2* e-preciso-adequar 3 colocar-o-peito 4 cuidar-de-mim 5 insatisfeita
039; 1 insatisfeita 2 tristeza 3 peito-pequeno 4* insatisfeita 5 fazer-adequacao-genital
040; 1 felicidade 2 bem-estar-comigo-mesma 3* satisfeita 4 felicidade 5 satisfeita
041; 1 insatisfeita 2 e-preciso-adequar 3 saudavel 4* peito-pequeno 5 insatisfeita
042; 1 e-preciso-adequar 2 saudavel 3 cuidar-de-mim 4* peito-pequeno 5 bem-estar-comigo-mesma
043; 1* insatisfeita 2 e-preciso-adequar 3 saudavel 4 e-preciso-adequar 5 tristeza
044; 1* saudavel 2 insatisfeita 3 insatisfeita 4 e-preciso-adequar 5 harmonizacao-facial
045; 1 saudavel 2 cuidar-de-mim 3 respeito-comigo-mesma 4* e-preciso-adequar 5 bem-estar-comigo-mesma
046; 1 saudavel 2 bem-estar-comigo-mesma 3 cuidar-de-mim 4* e-preciso-adequar 5 fazer-adequacao-genital
047; 1 bem-estar-comigo-mesma 2* e-preciso-adequar 3 saudavel 4 bonito 5 fazer-adequacao-genital
048; 1* e-preciso-adequar 2 peito-pequeno 3 fazer-adequacao-genital 4 harmonizacao-facial 5 bunda-pequena
049; 1* satisfeita 2 e-preciso-adequar 3 saudavel 4 bem-estar-comigo-mesma 5 cuidar-de-mim
050; 1* e-preciso-adequar 2 e-preciso-adequar 3 insatisfeita 4 cuidar-de-mim 5 feminilidade
051; 1 saudavel 2 bem-estar-comigo-mesma 3 e-preciso-adequar 4 tristeza 5* bem-estar-comigo-mesma
052; 1 satisfeita 2 viver 3 viver 4 viver 5* viver
053; 1* tristeza 2 insatisfeita 3 e-preciso-adequar 4 colocar-o-peito 5 e-preciso-adequar
054; 1 saudavel 2* cuidar-de-mim 3 colocar-o-peito 4 fazer-adequacao-genital 5 respeito-comigo-mesma
055; 1 e-preciso-adequar 2 viver 3* saudavel 4 fazer-adequacao-genital 5 cuidar-de-mim
056; 1 cuidar-de-mim 2* bem-estar-comigo-mesma 3 e-preciso-adequar 4 saudavel 5 felicidade

057; 1 bonito 2 insatisfeita 3 tristeza 4* uma-prisao 5 tristeza
058; 1 insatisfeita 2 insatisfeita 3* saudavel 4 bem-estar-comigo-mesma 5 satisfeita
059; 1 tristeza 2 tristeza 3 bonito 4* insatisfeita 5 bem-estar-comigo-mesma
060; 1 satisfeita 2 bem-estar-comigo-mesma 3* respeito-comigo-mesma 4 respeito-comigo-mesma 5 bonito
061; 1 cuidar-de-mim 2 satisfeita 3 e-preciso-adequar 4 e-preciso-adequar 5* colocar-o-peito
062; 1 uma-prisao 2 tristeza 3 saudavel 4 e-preciso-adequar 5* e-preciso-adequar
063; 1 felicidade 2* felicidade 3 satisfeita 4 feminilidade 5 felicidade
064; 1 insatisfeita 2 insatisfeita 3* e-preciso-adequar 4 colocar-o-peito 5 saudavel
065; 1 felicidade 2* colocar-o-peito 3 saudavel 4 cuidar-de-mim 5 respeito-comigo-mesma
066; 1 e-preciso-adequar 2 saudavel 3 tristeza 4* esta-velho 5 bem-estar-comigo-mesma
067; 1 satisfeita 2 satisfeita 3 satisfeita 4 satisfeita 5* cuidar-de-mim
068; 1 ganhar-dinheiro 2 respeito-comigo-mesma 3 respeito-comigo-mesma 4* respeito-comigo-mesma 5 insatisfeita
069; 1 satisfeita 2 felicidade 3 felicidade 4 felicidade 5* respeito-comigo-mesma
070; 1* insatisfeita 2 respeito-comigo-mesma 3 uma-prisao 4 e-preciso-adequar 5 felicidade
071; 1* felicidade 2 satisfeita 3 cuidar-de-mim 4 satisfeita 5 felicidade
072; 1 colocar-o-peito 2 fazer-adequacao-genital 3 saudavel 4 tristeza 5* viver
073; 1 bonito 2 bonito 3* felicidade 4 cuidar-de-mim 5 cuidar-de-mim
074; 1 bonito 2 bonito 3 bonito 4 bonito 5* colocar-o-peito
075; 1* e-preciso-adequar 2 bem-estar-comigo-mesma 3 saudavel 4 cuidar-de-mim 5 fazer-adequacao-genital
076; 1 insatisfeita 2 harmonizacao-facial 3 praia 4 cuidar-de-mim 5* tristeza
077; 1 e-preciso-adequar 2 e-preciso-adequar 3 cuidar-de-mim 4 cuidar-de-mim 5* ganhar-dinheiro
078; 1 colocar-o-peito 2* bonito 3 bonito 4 bonito 5 bonito
079; 1 felicidade 2* felicidade 3 felicidade 4 respeito-comigo-mesma 5 satisfeita
080; 1 feminilidade 2 feminilidade 3 uma-prisao 4 ganhar-dinheiro 5* uma-prisao
081; 1 bonito 2 e-preciso-adequar 3 harmonizacao-facial 4 harmonizacao-facial 5* bonito
082; 1* saudavel 2 satisfeita 3 felicidade 4 satisfeita 5 ganhar-dinheiro
083; 1 e-preciso-adequar 2 e-preciso-adequar 3 e-preciso-adequar 4 saudavel 5* cuidar-de-mim
084; 1 tristeza 2 insatisfeita 3* uma-prisao 4 medo-de-envelhecer 5 e-preciso-adequar
085; 1 e-preciso-adequar 2* respeito-comigo-mesma 3 respeito-comigo-mesma 4 feminilidade 5 bonito
086; 1 felicidade 2 bonito 3* felicidade 4 respeito-comigo-mesma 5 bonito
087; 1* peito-pequeno 2 insatisfeita 3 insatisfeita 4 harmonizacao-facial 5 harmonizacao-facial
088; 1* tristeza 2 estranho 3 tristeza 4 tristeza 5 insatisfeita
089; 1* felicidade 2 satisfeita 3 satisfeita 4 uma-prisao 5 harmonizacao-facial
090; 1 satisfeita 2 bonito 3 bonito 4 bem-estar-comigo-mesma 5* saudavel
091; 1 bonito 2 insatisfeita 3 estranho 4* bonito 5 bonito
092; 1* viver 2 feminilidade 3 insatisfeita 4 bem-estar-comigo-mesma 5 bonito
